# Sensitive voltammetric detection of bosentan using gold nanoparticles-decorated pencil graphite electrode in pharmaceutical formulations and plasma samples

**DOI:** 10.1038/s41598-026-42667-w

**Published:** 2026-03-27

**Authors:** Yossra A. Trabik, Reham A. Ismail, Miriam F. Ayad, Lobna A. Hussein, Amr M. Mahmoud

**Affiliations:** 1 Pharmaceutical Analytical Chemistry Department, Faculty of Pharmacy, Ain Shams University, Organization of African Unity Street, Abassia, Cairo, 11566 Egypt; 2 Analytical Chemistry Department, Faculty of Pharmacy, Cairo University, El-Kasr El-Aini Street, Cairo, 11562 Egypt

**Keywords:** Bosentan, Differential pulse voltammetry, Pencil graphite electrode, Gold nanoparticles, Pharmaceutical dosage form, Human plasma, Chemistry, Nanoscience and technology

## Abstract

In this study, a new eco-friendly electrochemical sensor was effectively designed and utilized to analyze bosentan (BOS); an endothelin receptor antagonist (ERA) that helps in the management of pulmonary artery hypertension (PAH), in its pharmaceutical dosage forms and in human plasma samples. Our approach involves BOS electro-oxidation on a gold nanoparticles- modified pencil graphite electrode (PGE) surface in order to improve electrode sensitivity and increase peak current. Gold nanoparticles (Au-NPs) were electrochemically deposited onto the electrode surface using cyclic voltammetry (CV). With the aid of differential pulse voltammetry, a quantitative determination of BOS was obtained in 0.1 M KCl across a potential range of 0.00 to 1.8 V. The method’s validation was performed in compliance with International Council for Harmonization of Technical Requirements for Pharmaceuticals for Human Use (ICH) guidelines, and the dynamic range of the proposed sensor was found to be (2.5 × 10^− 7^–2.5 × 10^− 5^) M with a limit of detection (LOD) of (7.92 × 10^− 8^) M. The sensor has been effectively utilized to determine BOS in its dosage form and human plasma with good accuracy and precision, and without any interference from the plasma matrix or tablet excipients. In addition, the method was assessed from a green perspective and was found to be of excellent greenness with an analytical eco-scale of 78.0. While AGREE software gave a score of 0.65 which indicates acceptable greenness.

**Supplementary Information:**

The online version contains supplementary material available at 10.1038/s41598-026-42667-w.

## Introduction

**Fig. 1:**
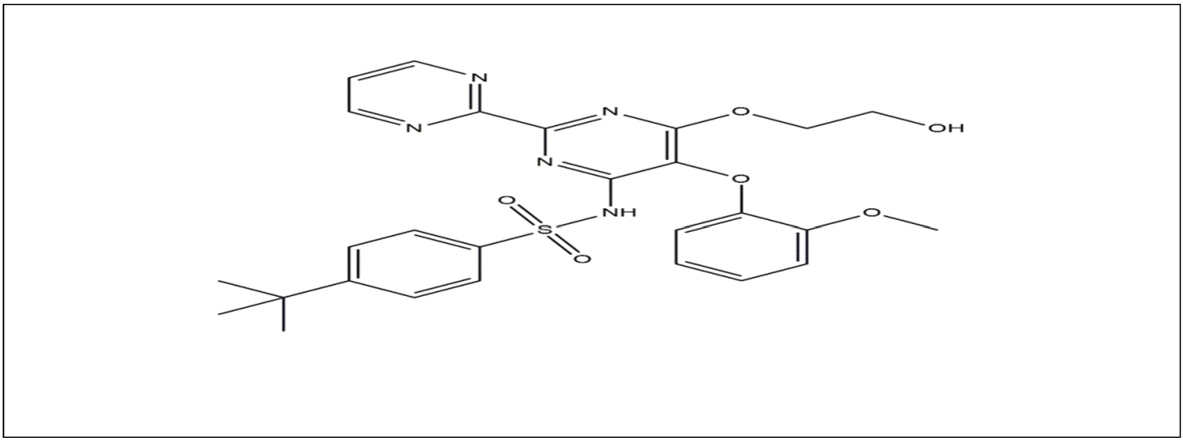
Chemical structure of bosentan (drawn by chemibio draw ultra).

Bosentan (BOS), also known as 4-tert-butyl-N-[6-(2-hydroxyethoxy)-5-(2-methoxyphenoxy)-2-(pyrimidin-2-yl) pyrimidin-4-yl] benzene sulfonamide (Fig. 1), is an oral antagonist of endothelin A and B receptors. It is part of a significant class of medications that are used to treat pulmonary artery hypertension (PAH), a condition in which high levels of endothelin, a powerful blood vessel constrictor, have been detected in the plasma and lung tissue of patients with PAH, which results in a mean pulmonary artery pressure (mPAP) of 25 mmHg or higher at rest^1^. BOS is the first orally active medication licensed by the U.S Food and Drug Administration (FDA) in 2001 for the treatment of PAH. It alleviates symptoms, particularly in those with World Health Organization (WHO) Class III or IV symptoms, by vasodilation, antifibrotic, and antithrombotic effects^2–4^. Because of its antiviral properties, BOS may potentially be used medicinally to treat COVID-19 when taken in conjunction with other authorized medications^5,6^.

Numerous analytical methods, including potentiometry^7^, spectroscopy and chromatography, have been published to determine BOS in biological fluids and pharmaceutical dosage forms^8–13^. The only voltammetric method employed for the examination of BOS in pharmaceutical preparation is based on the electrochemical oxidation of BOS at a platinum electrode using linear sweep, square wave, and differential pulse voltammetry^14^, but no voltammetric methods have been reported for the analysis of BOS in human plasma.

Analytical and electroanalytical methods are essential for sensitive, selective, and rapid analysis of chemical substances, enabling trace-level detection with low cost and minimal sample preparation. Advances in electrochemical techniques and electrode materials have improved analytical performance and provided valuable insight into redox mechanisms, making these methods indispensable in modern research and applied sciences^15,16^.

Regarding the investigation of various inorganic and organic substances, voltammetric methods are receiving a lot of attention due to their simplicity, high sensitivity^17^, broad linear range, low cost, and quick analysis time^18^, in addition to traditional glassy carbon electrode a number of carbonaceous substrates, including screen-printed electrodes, microfabricated electrodes, boron-doped diamond, paper-based electrodes, and pencil graphite electrodes (PGEs), have been studied for use in the fabrication of electrochemical sensors^19^.

Our study is concerned with PGEs, as they are widely commercially available in the market, have low background current, are easily modifiable, inexpensive, mechanically robust, disposable, avoid the tedious process of polishing the surface of solid electrodes in between measurements, and their surface modification is a simple process that enables the measurement of low concentrations^20,21^. Graphene-like sheets such as molybdenum disulfide nanosheets, conducting polymers, metal oxide nanoparticles, metal-organic frameworks, graphene and carbon nanotubes, and molecularly imprinted polymers have all been used to modify PGEs in recent years. Additionally, because of their exceptional chemical, optical, electrical, and electrocatalytic qualities, gold nanoparticles have drawn a lot of interest in the field of electrochemistry. They can be used in PGEs modification using electrochemical techniques like cyclic voltammetric (CV) and chronoamperometric methods with a chloroauric acid solution, thus it is simple to modify PGEs with Au-NPs in situ. By optimizing the experimental parameters, such as the number of cycles, scan rate, and potential range, the size of the Au-NPs can be controlled^22^.

The synergistic combination of Au-NPs with PGE leads to improved signal-to-noise ratio, sharper voltammetric peaks, reduced charge-transfer resistance, and lower limits of detection compared to bare electrode. So, the combined effect of the two materials creates a powerful, simple and inexpensive, sensing platform with better activity and sensitivity^23–25^.

In this work, we intend to use differential pulse voltammetric approach (DPV) for the sensitive assessment of BOS in human plasma and pharmaceutical dosage forms utilizing a pencil graphite electrode modified with gold nanoparticles (PGE/Au-NPs). To comprehend the electrochemical behavior of BOS, the effect of scan rate was investigated and optimized. To demonstrate how Au-NPs affect electrode sensitivity, different electrodes including glassy carbon, bare PGE, and PGE/Au-NPs, were compared.

## Experimental

### Material and reagents

A certified standard of bosentan (BOS) was generously donated by EVA Pharma (Cairo, Egypt). Its purity was measured using the described HPLC method^13^, and the results showed that it was 99.83 ± 0.45. Methanol of HPLC grade was bought from Fisher Scientific (UK). Chloroauric acid (HAuCl_4_) was purchased from Sigma Aldrich (Germany). KCl was purchased from El Nasr Company (Cairo, Egypt). Graphite pencils were acquired from a nearby bookstore, pencil leads made by Dong-A (HB), batch number/barcode (8802203018194), having a diameter of 0.90 mm and a length of 60.00 mm. Human plasma samples were purchased from VACSERA (Giza, Egypt) and stored at -4 °C. Pulmiprove^®^ 62.5 mg Tablets (batch number 2031657) was acquired from the local market, Cairo, Egypt.

### Instrumentation

Voltammetric measurements were performed using a Metrohm Computrace voltammetric analyzer (model 884 Professional VA, Switzerland) equipped with a type III potentiostat (l-AUTO LAB). A three electrodes configuration was used: a platinum wire as the auxiliary electrode, glassy carbon electrode (GCE, Ø2.0 mm), bare pencil graphite electrode, and modified pencil graphite electrode with gold nanoparticles (PGE/Au-NPs) as the working electrodes, and an Ag/AgCl reference electrode (3 M KCl). A PC connected to an 884 Professional VA Computrace electrochemical analyzer was used to process the data.

#### Working electrode

Disposable pencil graphite electrodes coated with gold nanoparticles (PGE/Au-NPs) were eventually used. Every measurement in the measuring procedure was made using a fresh new PGE.

#### Gold nanoparticles deposition on PGE

**Fig. 2:**
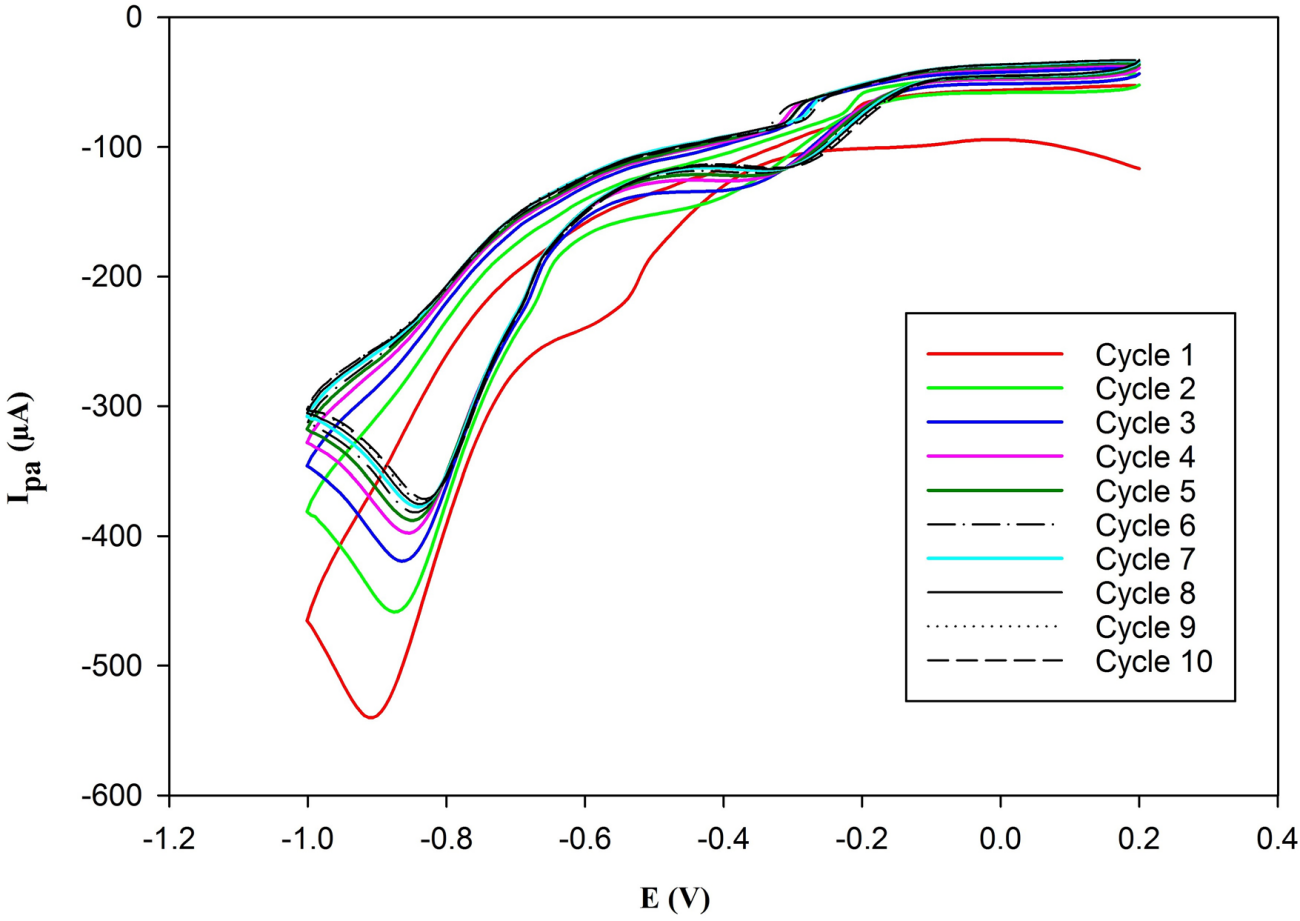
Cyclic volammograms of gold nanoparticles deposition on PGE/Au-NPs electrode using 5 × 10^–4^ M HAuCl_4_ in 0.1 M KCl as supporting electrolyte, and scan rate of 50 mV/s.

The gold nanoparticles deposition was performed as reported in the literature^19^ and illustrated in Fig. 2. After immersing the PGE in 5.0 × 10^− 4^ M HAuCl_4_ in 0.1 M KCl as a supporting electrolyte, cyclic voltammetry (CV) was carried out. The potential scanning started at + 0.2 V to − 1.0 V vs. an Ag/AgCl reference electrode and continued for 10 cycles at a scan rate (υ) of 50 mV/s.

### BOS standard stock solution

A stock solution of BOS (1.0 × 10^− 4^) M was prepared by weighing 5.52 mg of BOS into a 100.0 mL volumetric flask, which was then dissolved in an adequate quantity of methanol before filling it up to the final volume with the same solvent.

### BOS working solution

By gradually diluting the BOS stock solution with 0.1 M KCl as a supporting electrolyte, new solutions with varying strengths (2.5 × 10^− 7^–2.5 × 10^− 5^) M were obtained.

### Electrochemical measurements and calibration curve construction

By utilizing cyclic voltammetry (CV), the electrochemical behavior of BOS was examined at the PGE/Au-NPs electrode in 0.1 M KCl as the supporting electrolyte. A representative cyclic voltammogram of 2.5 × 10^− 5^ M BOS was acquired under a scan rate of 50.0 mV/s and a potential range of 0 to 2.0 V. For quantitative analysis of BOS, differential pulse voltammograms (DPVs) were recorded using PGE/Au-NPs vs. the Ag/AgCl reference electrode (3.0 M KCl) for each concentration while applying a scan rate (υ) of 50.0 mV/s, a pulse amplitude of 50.0 mV, measuring time = 0.02 s, potential step time = 0.20 s, and a pulse width of 40.0 ms during scanning potentials ranging from 0.00 to 1.8 V. The peak current height (I_p.a._) for each sample was plotted against the corresponding concentration to create the calibration curve.

### Effect of scan rate

The impact of scan rate (υ) on the peak current height I_p.a._ was examined using CV. The cyclic voltammograms were recorded at various scan rates, which ranged from 20.00 mV/s to 500.00 mV/s. Investigation of the mass transfer process involved evaluating the relationship between log I_p.a._ and log υ.

### Method validation

The suggested approach was verified based on ICH criteria^26^ concerning linearity, limit of detection (LOD), limit of quantification (LOQ), accuracy, and precision. Linearity was determined by transferring precisely measured volumes of BOS stock solution into a set of 50 mL volumetric flasks, and a series of dilutions ranging from (2.50 × 10^− 7^–2.50 × 10^− 5^) M, were created independently. Differential pulse voltammograms were recorded using the same conditions mentioned in section “Electrochemical measurements and calibration curve construction”. for each dilution. Then accuracy was determined by measuring percentage recoveries of five different concentrations of pure sample of BOS (2.0 × 10^− 6^, 7.0 × 10^− 6^, 1.20 × 10^− 5^, 1.35 × 10^− 5^, and 1.50 × 10^− 5^) M three times. Precision was estimated as relative standard deviation (%RSD) by measuring three different concentrations of pure samples of BOS (2.0 × 10^− 6^, 7.0 × 10^− 6^ and 1.2 × 10^− 5^) three times on the same day (Repeatability) and on three consecutive days (Reproducibility). LOD and LOQ were calculated using the following formulas:

$${\mathrm{LOD}}\,=\,{\mathrm{3}}.{\text{3 }} \times \sigma /{\mathrm{S}}$$

$${\mathrm{LOQ}}\,=\,{\mathrm{1}}0{\text{ }} \times \sigma /{\mathrm{S}}$$

where S is the slope of the calibration curve for the drug under study, and σ is the standard deviation of the intercept.

### BOS analysis in the pharmaceutical tablet formulation

A total of ten tablets of BOS in its pharmaceutical preparation (Pulmiprove ^®^ tablets) were precisely weighed and finely powdered. Powder equivalent to 2.7 mg of BOS was transferred to a 50 mL volumetric flask. The volume was then filled with methanol and was shaken for approximately 10 min, and the contents were filtered through Whatman filter paper No. 42 to prepare 1.0 × 10^− 4^ M BOS stock solution. The filtered mixture was suitably diluted using the corresponding solvent to achieve varying concentrations of (1.5 × 10^− 6^, 5.5 × 10^− 6^, and 2.0 × 10^− 5^) M. Differential pulse voltammograms were acquired for the prepared solutions using the previously optimized parameters .

### BOS analysis in spiked human plasma

For protein precipitation, different volumes of BOS stock solution were added to 1.0 mL of human plasma in a set of Eppendorf tubes to produce the following concentrations: (1.0 × 10^− 6^, 5.0 × 10^− 6^, and 2.0 × 10^− 5^) M including the highest plasma concentration (C _max_)^27^. Next, 4.0 mL of methanol was added to precipitate the plasma proteins. The tubes were centrifuged at 6,000 rpm for 20 min. After that, the supernatant was dried by evaporation and reconstituted with 0.1 M KCl in a 10.0 mL volumetric flask. Differential pulse voltammograms were obtained using the previously optimized parameters.

## Results and discussion

### Characterization

The surface morphology of PGE/Au-NPs has been characterized in our recent publication^28^ using both scanning electron microscopy (SEM) and X-ray photoelectron Spectroscopy (XPS) as shown in Fig. S1.

### Voltammetric behavior of BOS

**Fig. 3:**
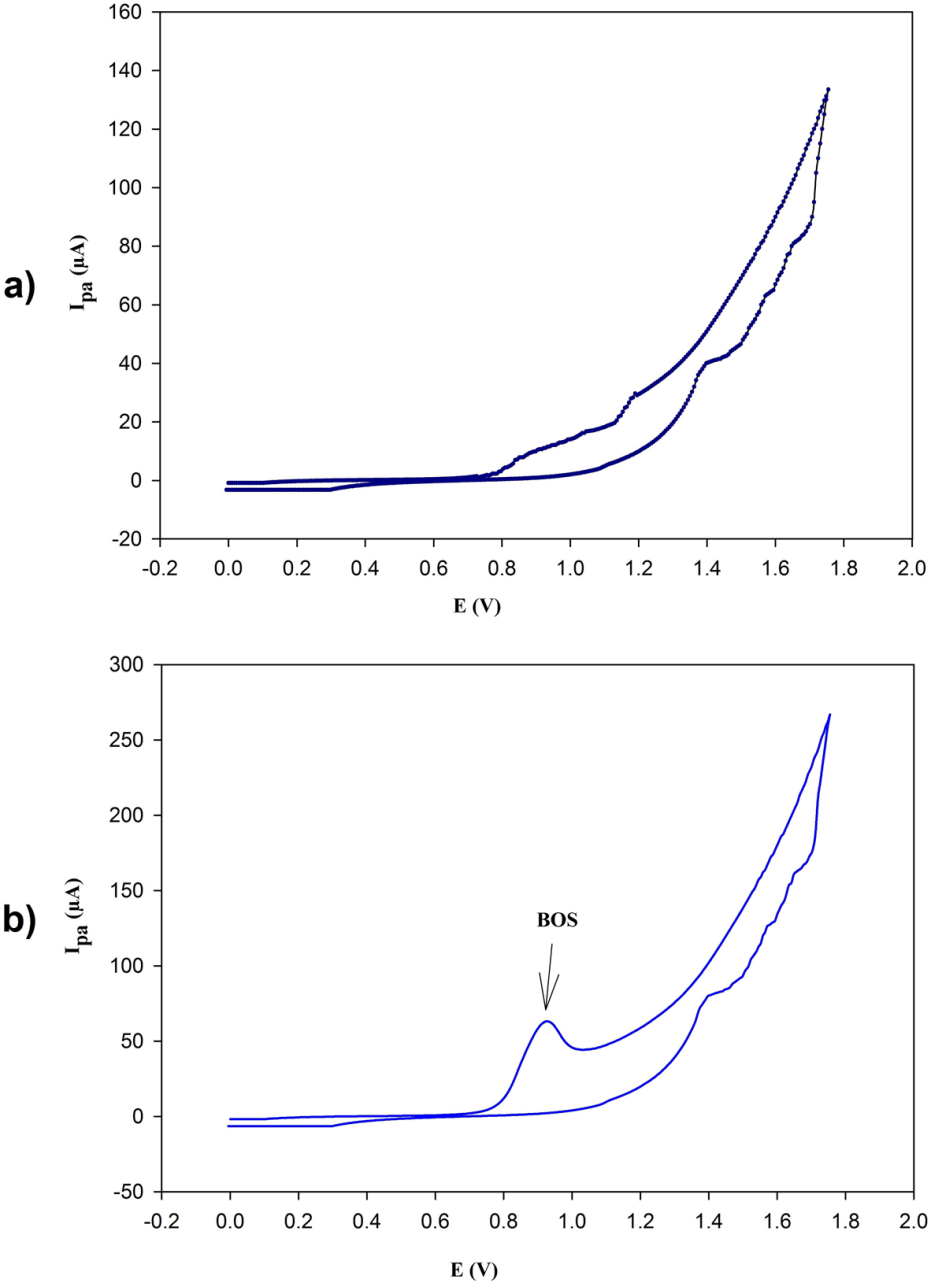
(a) Cyclic voltammogram of 0.1 M KCl without BOS using PGE/Au-NPs electrode with scan rate 50.0 mV/s. (b) Cyclic voltammogram of 2.5 × 10^–5^ M of BOS using PGE/Au-NPs electrode with scan rate 50.0 mV/s, showing anodic peak at 0.975 V.

By utilizing CV, the electrochemical behavior of BOS was examined at the PGE/Au-NPs electrode in 0.1 M KCl as the supporting electrolyte. A representative cyclic voltammogram of 2.5 × 10^− 5^ M BOS was acquired under a scan rate of 50 mV/s and a potential range of 0 to 2.0 V, as shown in Fig. 3. At a potential of around + 0.975 V, an oxidation peak was observed in the anodic sweep. This oxidation wave’s accompanying reduction peak is absent when the potential scan is reversed, demonstrating the irreversible nature of the electrode process.

**Scheme 1:**
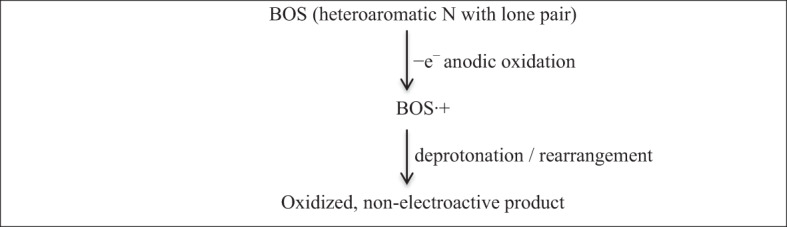
The proposed mechanism of BOS oxidation at PGE/Au-NPs electrode.

BOS contains several functional groups, including sulfonamide, aromatic rings, and heteroaromatic nitrogen centers. Sulfonamide groups are well known for their electrochemical stability and typically undergo oxidation only at significantly higher potentials than those applied in this study^29^. Therefore, they are unlikely to contribute to the observed anodic response. In contrast, heteroaromatic nitrogen atoms possess lone pair of electrons that are conjugated with aromatic systems, making them favorable sites for anodic oxidation^30^. Accordingly, the oxidation of BOS is assigned to a one-electron transfer occurring at an electron-rich heteroaromatic nitrogen center, resulting in the formation of a nitrogen-centered radical cation. This intermediate is highly unstable and rapidly undergoes a chemical transformation, such as deprotonation or intramolecular rearrangement, yielding a non-electroactive product (Scheme 1). The fast follow-up chemical step suppresses any reverse reduction process, thereby explaining the irreversible voltammetric behavior.

**Fig. 4:**
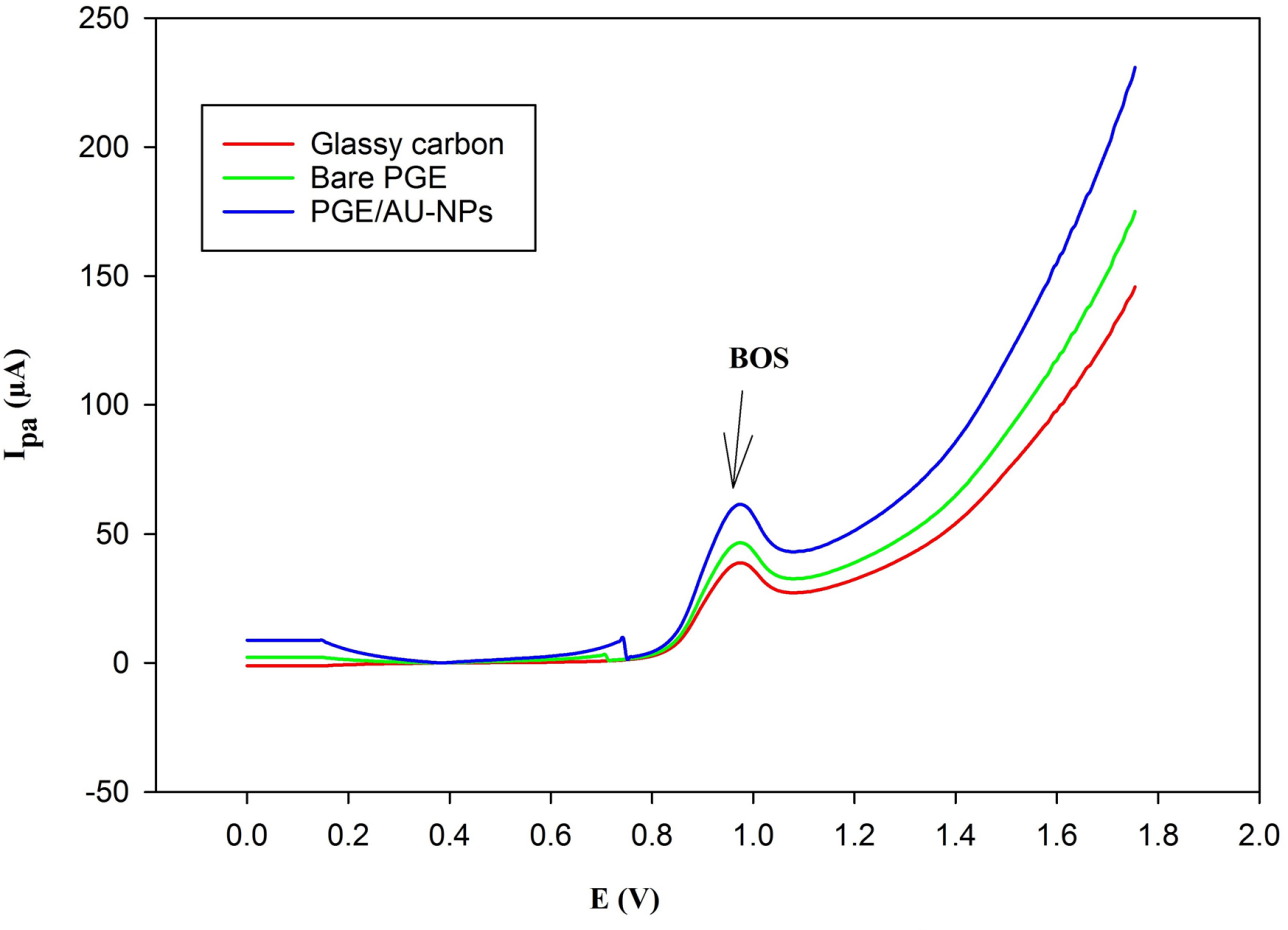
Differential pulse voltammogram of 5.0 × 10^–6^ M of BOS using: Glassy carbon, Bare PGE, and PGE/Au-NPs electrode with scan rate of 50 mV/s, showing anodic peak at 0.975 V.

In order to prove the effect of Au-NPs on peak current and sensitivity, DPV on different working electrodes, including glassy carbon, bare PGE, and PGE/Au-NPs was acquired in the presence of 5.0 × 10^− 6^ of BOS in 0.1 M KCl, as shown in Fig. 4. The PGE/Au-NPs electrode showed a higher current response, with measured currents of 37 µA for 5.0 × 10^− 6^ M BOS on glassy carbon, 46.59 µA for bare PGE, and 65.72 µA with PGE/Au-NPs. The outcome demonstrates how the nanoparticles improved the peak current, due to the catalytic activity of Au-NPs and the increased electrode surface area, which are responsible for the current peak rise from 46.59 µA to 65.72 µA following PGE modification with Au-NPs. Therefore the PGE/Au-NPs electrode was the optimum choice for this investigation. The effect of acetate buffer and Britton–Robinson buffer as supporting electrolytes on the electrochemical response of BOS was investigated. However, neither electrolyte provided a noticeable improvement compared to KCl. As presented in Figures S2 and S3, both buffers exhibited lower current intensities and broader anodic peaks, suggesting less favorable electron-transfer conditions than those observed with KCl. BOS was detected electrochemically by oxidation at the PGE/Au-NPs surface in 0.1 M KCl at + 0.975 V. The DPV technique offers several advantages over previously reported potentiometric technique^7^ including enhanced selectivity, dual analysis (both qualitative and quantitative), and multiple species analysis.

### Effect of scan rate

**Fig. 5:**
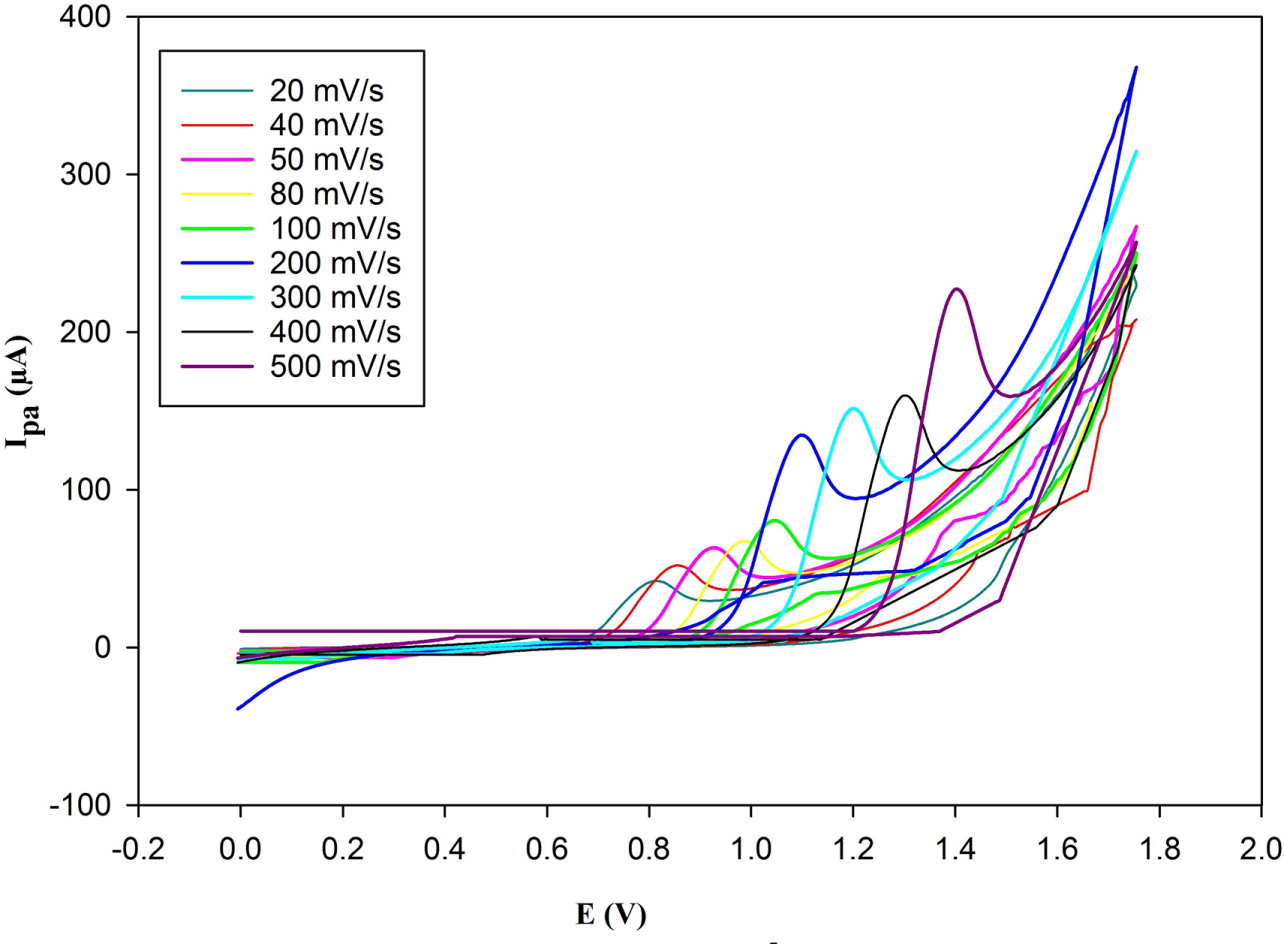
Cyclic voltammograms of 2.5 × 10^–5^ M of BOS using PGE/Au-NPs electrode as a function of scan rate (20.0–500.0 mV/s).

CV was used to examine the impact of scan rate on the anodic oxidation peak of 2.5 × 10^− 5^ M of BOS, over the range of (20.0–500.0) mV/s using the PGE/Au-NPs electrode, as illustrated in Fig. 5.

**Fig. 6:**
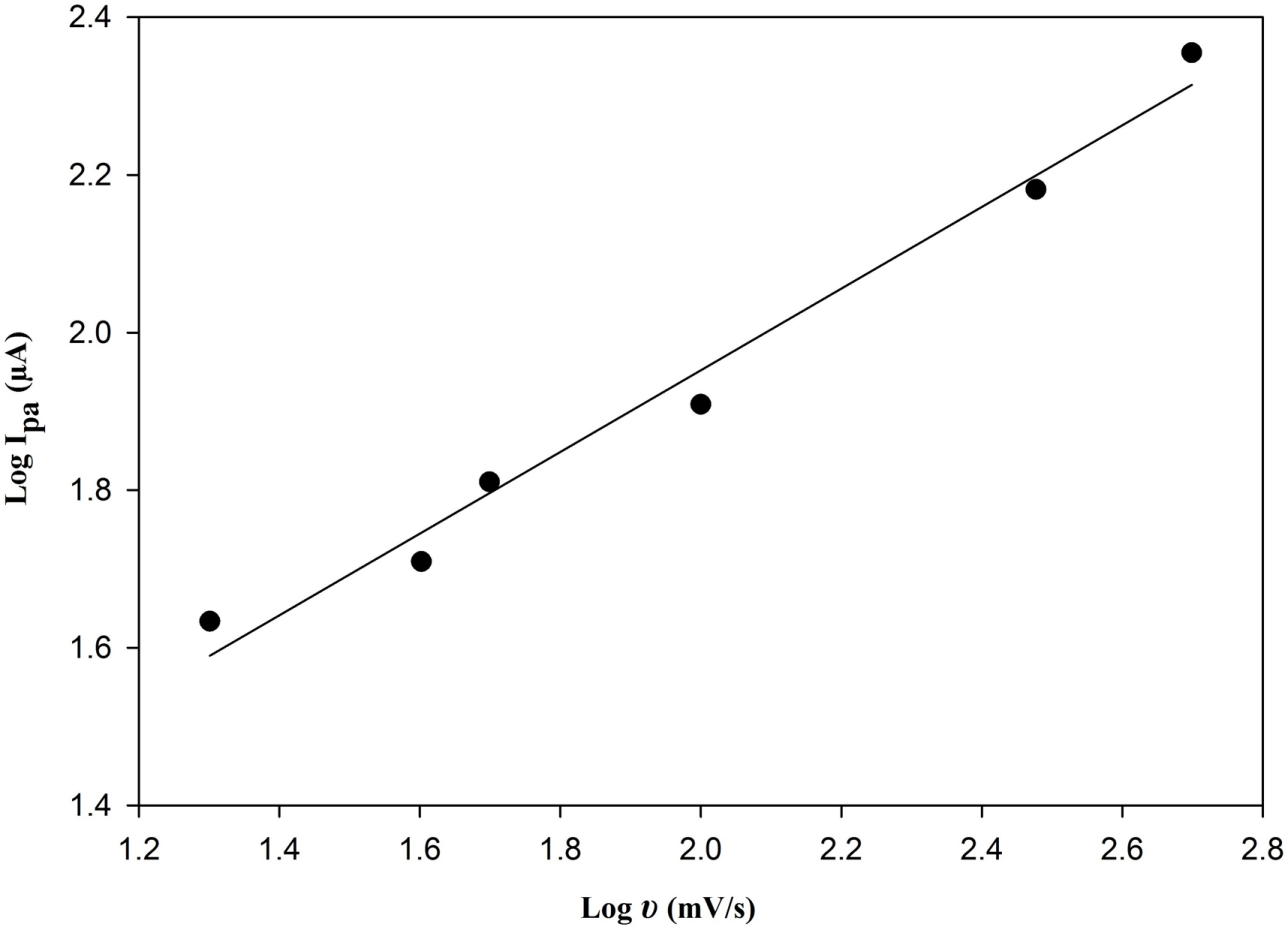
Relationship between log υ versus log I_pa_ for 2.5 × 10^–5^ M BOS at PGE/Au-NPs electrode.

The plot of log (I_p.a._) vs. log scan rate (υ) in Fig. 6 illustrates a straight line with the following regression equation:

$${\text{Log }}{{\mathrm{I}}_{{\mathrm{pa}}}}={\text{ }}0.{\mathrm{518}}0{\text{ Log}}\upsilon \,+\,0.{\mathrm{916}}0{\text{ }}({\mathrm{r}}\,=\,0.{\mathrm{99}}0{\mathrm{8}}).$$

The slope approaches the theoretical value of 0.5, that is anticipated for a perfect diffusion-controlled electrode process^31^, which is in compliance with the literature concerned with voltammetric determination of BOS^14^.

**Fig. 7:**
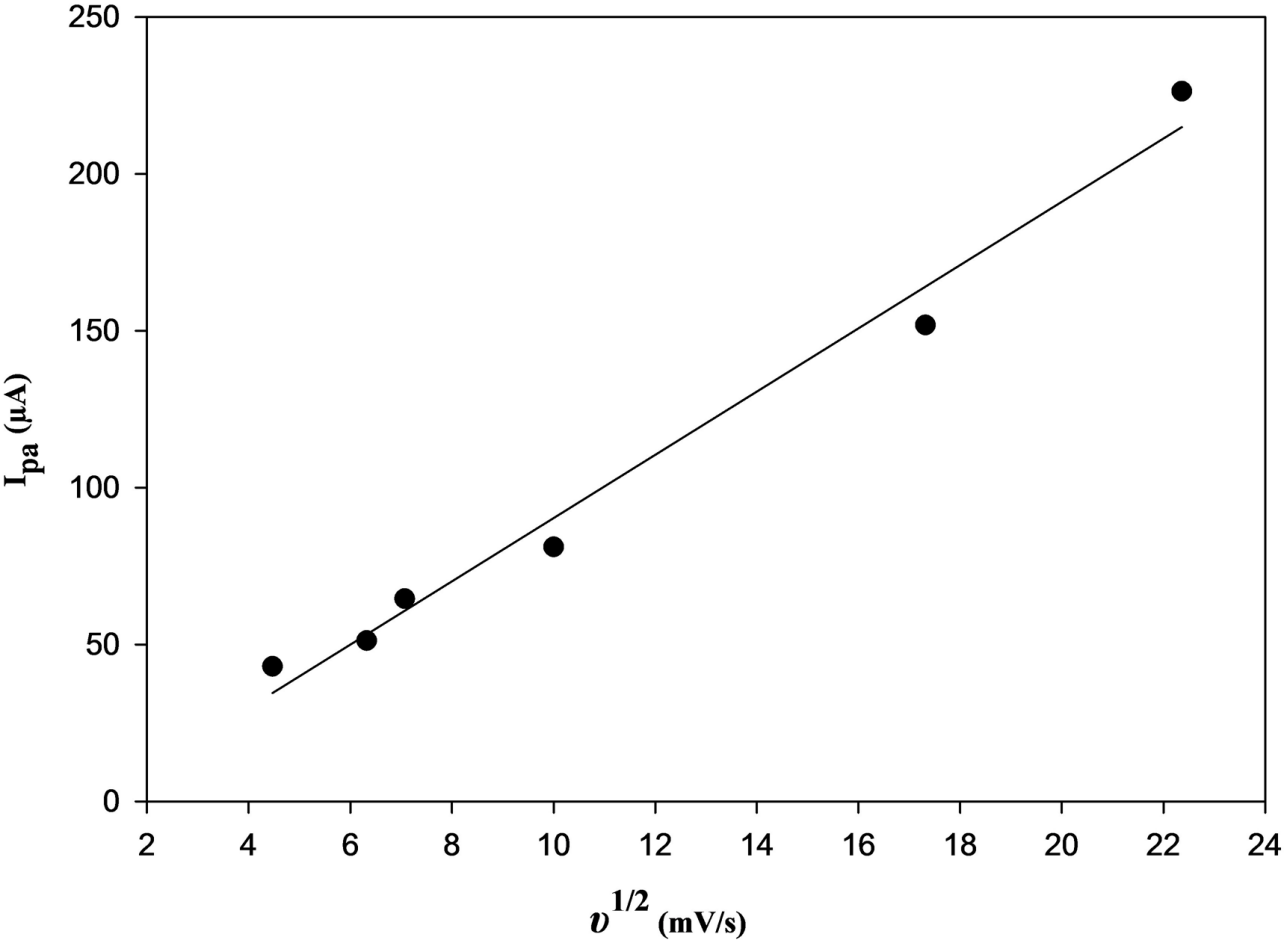
Relationship between υ^1/2^ versus I_pa_ for 2.5 × 10^–5^ M BOS at PGE/Au-NPs electrode.

Moreover, a direct proportionality was discovered between I_p.a._, and the square root of the scan rate (υ^1/2^) as indicated in Fig. 7, according to the equation:

$${{\mathrm{I}}_{{\mathrm{pa}}}}={\text{ 1}}0.0{\mathrm{868}}{\upsilon ^{{\mathrm{1}}/{\mathrm{2}}}}\, - \,{\mathrm{1}}0.{\text{5669 }}({\mathrm{r}}\,=\,0.{\mathrm{9911}}).$$

**Fig. 8:**
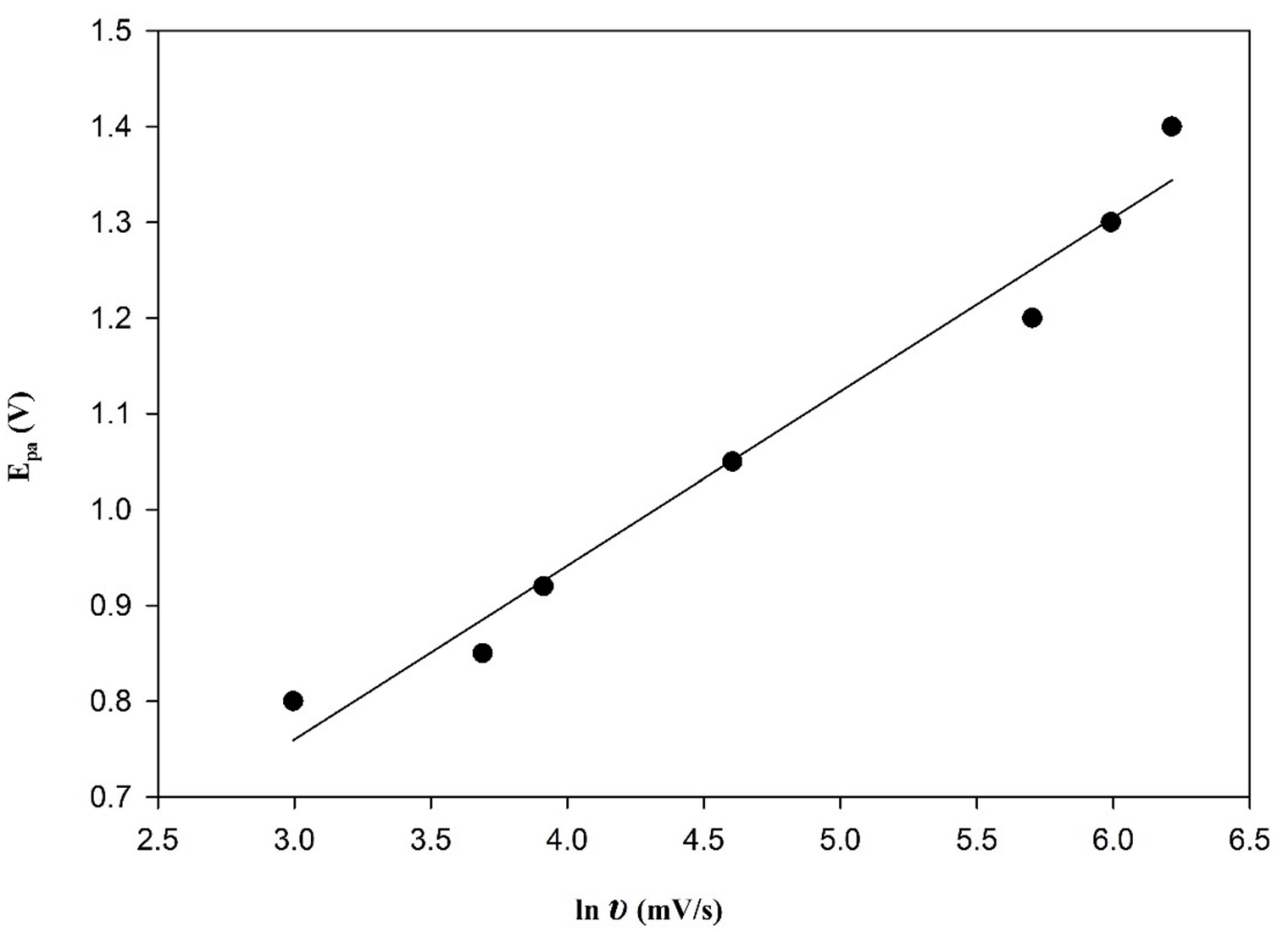
Relationship between ln υ versus E_pa_ for 2.5 × 10^–5^ M BOS at PGE/Au-NPs electrode.

Furthermore, as shown in Figs. 5 and 8, an increase in the scan rate value caused the peak potential to shift to a more positive value. A linear relationship between the peak potential (E_p.a._) and the natural logarithm of the scan rate (ln υ) was also established, indicating that the reaction is irreversible^32^, as previously proposed. The following is an illustration of the regression equation:

$${\mathrm{E}}_{{{\mathrm{pa}}}} = {\text{ }}0.{\text{1816 ln }}\upsilon {\text{ }} + {\text{ }}0.{\mathrm{2151}},{\text{ }}\left( {{\text{r }} = {\text{ }}0.{\mathrm{9864}}} \right).$$

To further characterize the kinetics of the electron transfer process for a totally irreversible electrode process, the dependence of the anodic peak potential (E_p.a._) on the scan rate (υ) is described by the equation proposed by Laviron as follows^33^:

$${\mathrm{E}}_{{{\mathrm{pa}}}} = {\mathrm{E}}^{0} + \,\left( {{\mathrm{RT}}/\alpha {\mathrm{nF}}} \right){\text{ ln }}\left( {{\mathrm{RTk}}^{0} /\alpha {\mathrm{nF}}} \right){\text{ }} - {\text{ }}\left( {{\mathrm{RT}}/\alpha {\mathrm{nF}}} \right){\text{ ln }}\upsilon$$

A linear relationship was obtained between (E_p.a._) and (ln υ), indicating the validity of Laviron’s model. Assuming a typical charge transfer coefficient (α) ≈ 0.5, *R* = 8.314 J mol^–1^ K^–1^, T = 298 K, and F = 96,485 C mol^–1^ for irreversible oxidation, the number of electrons involved in the rate-determining step was estimated to be approximately one. This result confirms that the oxidation of BOS proceeds via a single-electron transfer mechanism.

### Method validation

**Fig. 9:**
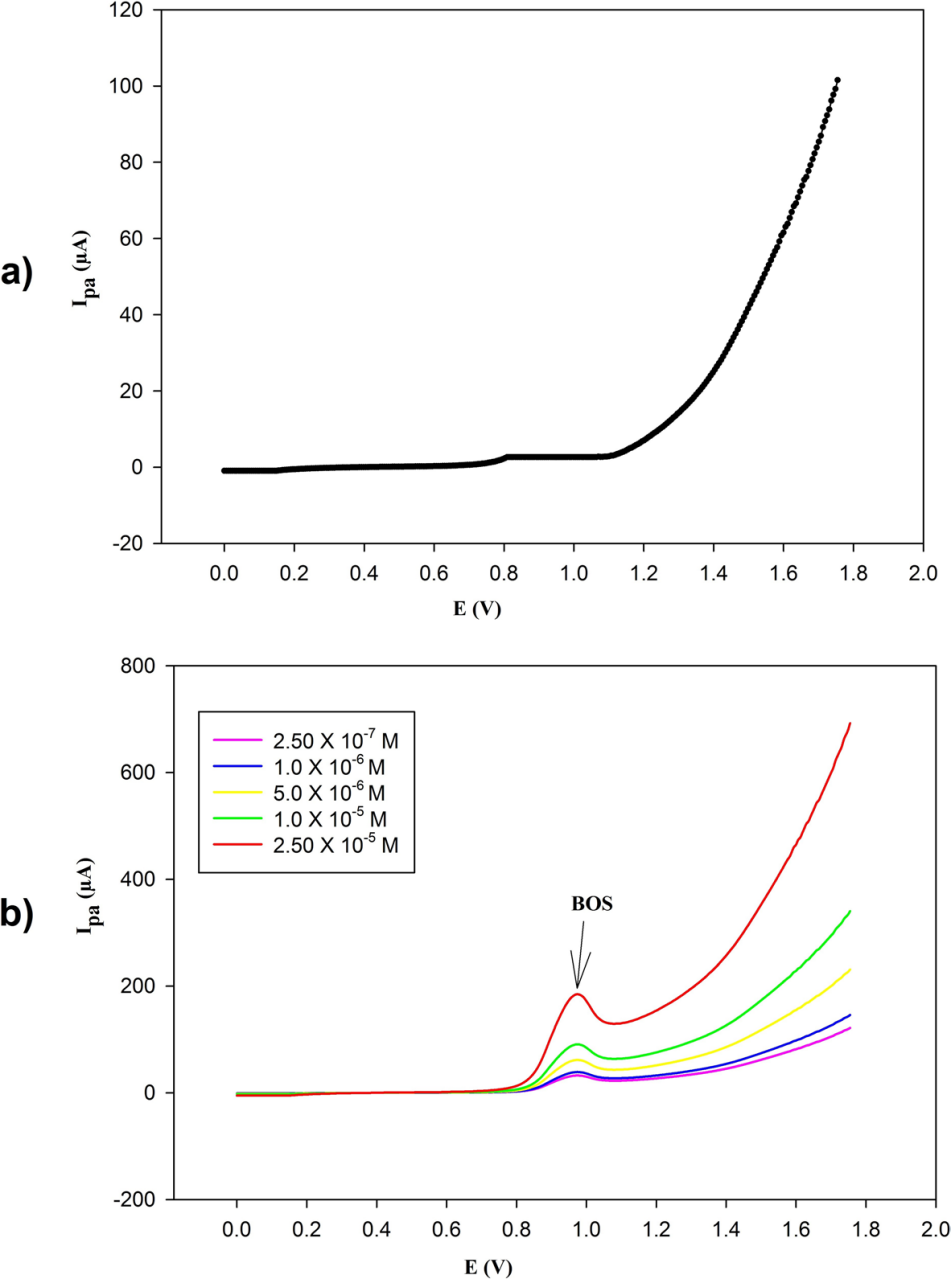
(a) Differential pulse voltammogram of 0.1 M KCl without BOS using PGE/Au-NPs electrode with scan rate of 50 mV/s. (b) Differential pulse voltammograms of BOS at different concentrations in the range of (2.5 × 10^–7^–2.5 × 10^–5^ M) BOS using PGE/Au-NPs electrode with scan rate of 50 mV/s, showing anodic peak at 0.975 V.

**Fig. 10:**
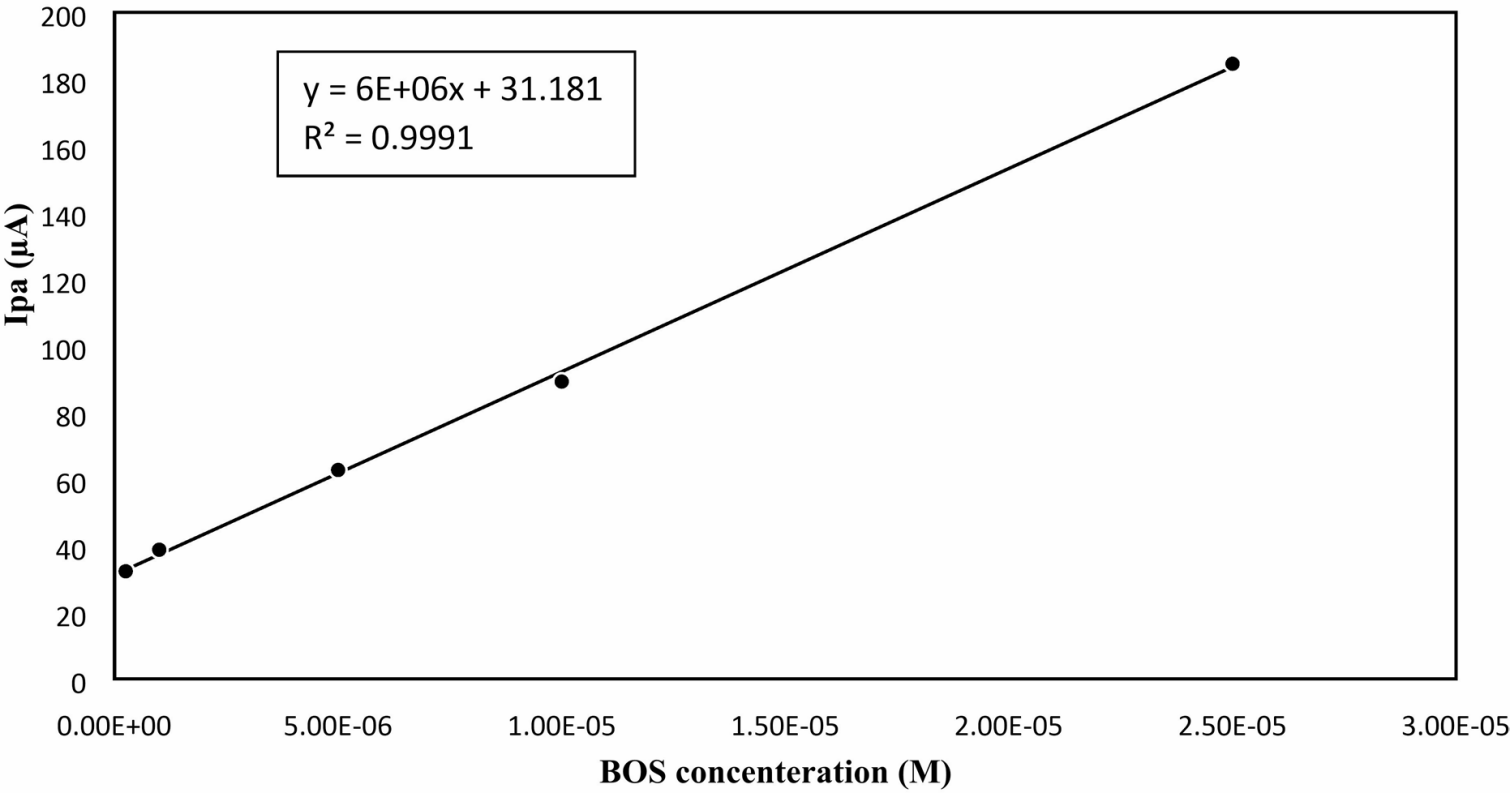
Calibration curve for determination of BOS in the range of (2.5 × 10^–7^–2.5 × 10^–5^ M) using PGE/Au-NPs electrode.

Under the optimum electrochemical conditions, the response was linear in the range of (2.50 × 10^− 7^–2.50 × 10^− 5^) M, with a correlation coefficient (r) of 0.9991 as illustrated in Figs. 9 and 10 and S4.

Table 1 presents a summary of the results of the calculation of various validation parameters in accordance with ICH criteria. Comparing the obtained results to the previously reported DPV method^14^, the proposed method showed around a tenfold increase in sensitivity compared to the reported DPV method, exhibiting a LOD of 7.92 × 10^− 8^ M. The accuracy of the analytical method showed that there is a strong agreement between the claimed and actual values as presented in Table 2. The proposed method is precise, as demonstrated by the good findings obtained from analyzing repeatability and reproducibility, where the % RSD values were less than 2%, as presented in Table 3.

**Table 1:** Parameters for the proposed DPV method for determination of BOS.

Parameter	BOS
Linearity rang (M)	2.50 × 10^− 7^–2.50 × 10^− 5^
Slope	6 × 10^6^
Intercept	31.181
Correlation coefficient (r)	0.9995
Accuracy * (mean ± SD)	99.35 ± 0.88
LOD (M)	7.92 × 10^− 8^
LOQ (M)	2.4 × 10^− 7^

*Average of five determinations.

**Table 2:** Accuracy results of the proposed DPV method for determination of BOS.

Claimed (M)	Found (M)	% Recovery *
2.00 × 10^− 6^	2.01 × 10^− 6^	100.50 ± 1.98
7.00 × 10^− 6^	6.89 × 10^− 6^	98.43 ± 1.12
1.20 × 10^− 5^	1.19 × 10^− 5^	99.17 ± 1.29
1.35 × 10^− 5^	1.35 × 10^− 5^	100.00 ± 0.67
1.50 × 10^− 5^	1.48 × 10^− 5^	98.67 ± 1.04
Mean ± S.D.		99.35 ± 0.88

* Average of three determinations.

**Table 3:** Precision results of the proposed DPV method for determination of BOS.

Claimed (M)	Repeatability*		Reproducibility**	
	Found (M)	% Recovery	Found (M)	% Recovery
2.00 × 10^− 6^	2.01 × 10^− 6^	100.50 ± 1.98	1.96 × 10^− 6^	98.00 ± 0.96
7.00 × 10^− 6^	6.95 × 10^− 6^	99.29 ± 1.34	6.68 × 10^− 6^	95.43 ± 0.71
1.20 × 10^− 5^	1.19 × 10^− 5^	99.17 ± 0.07	1.15 × 10^− 5^	95.83 ± 1.17
Mean ± S.D.	99.65 ± 0.736		96.42 ± 1.383	
%RSD (*n* = 9)	0.738		1.434	

* Repeatability: (*n* = 9), average of three concentration levels repeated three times within the same day.

** Reproducibility: (*n* = 9), average of three concentration levels repeated three times on three successive days.

### BOS analysis in the pharmaceutical tablet formulation

**Fig. 11:**
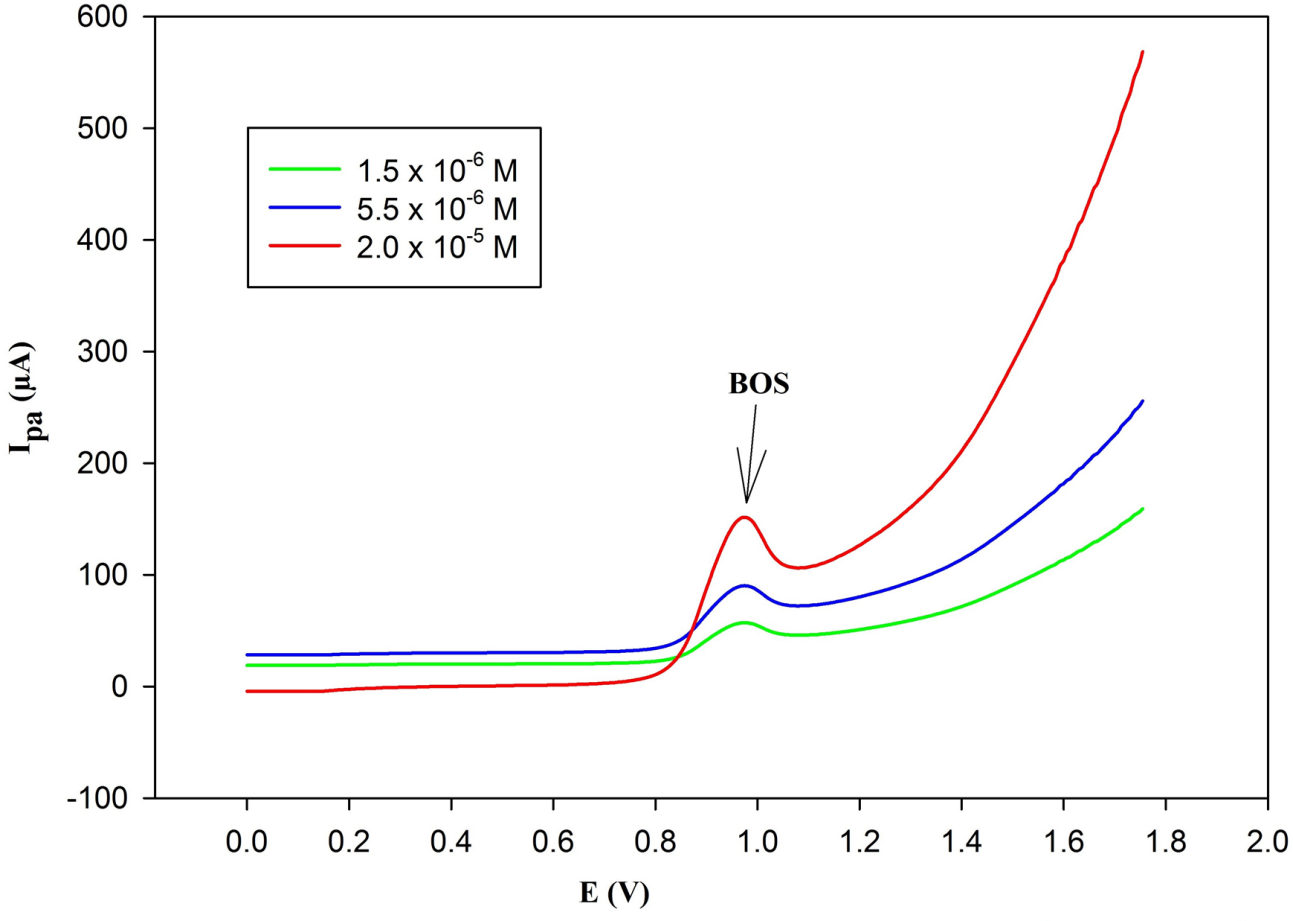
Differential pulse volammograms of (1.5 × 10^–6^, 5.5 × 10^–6^, 2.0 × 10^–5^ M) BOS in parmaceutical tablet formulation using PGE/Au-NPs electrode with scan rate of 50 mV/s, showing anodic peak at 0.975 V.

As illustrated in Fig. 11, a distinct anodic peak for BOS emerged at the chosen anodic potential (= 0.975 V against Ag/AgCl) at concentrations of (1.5 × 10^− 6^, 5.5 × 10^− 6^, and 2.0 × 10^− 5^) without any overlapping peaks from the excipients. The recoveries of BOS concentrations were found to be 100.42% ± 0.82, as presented in Table 4. It was discovered that the obtained results agreed with the claimed contents of BOS. No other component in the formulation had an impact on the analysis.

**Table 4:** Analysis of BOS in pharmaceutical tablet formulation.

Claimed (M)	Found (M)	% Recovery*
1.50 × 10^− 6^	1.51 × 10^− 6^	100.67 ± 1.61
5.50 × 10^− 6^	5.56 × 10^− 6^	101.09 ± 0.72
2.00 × 10^− 5^	1.99 × 10^− 5^	99.50 ± 0.40
Mean ± SD		100.42 ± 0.824

* Average of three determinations.

### BOS analysis in spiked human plasma

**Fig. 12:**
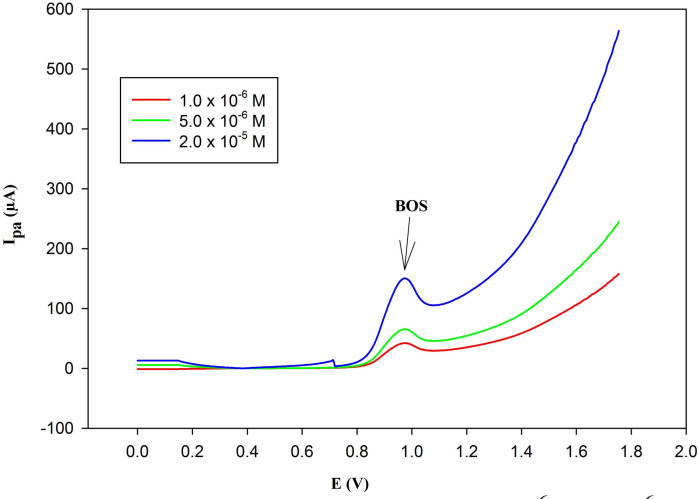
Differential pulse volammograms of (1.0 × 10^–6^, 5.0 × 10^–6^, 2.0 × 10^–5^ M) BOS in spiked human plasma using PGE/Au-NPs electrode with scan rate of 50 mV/s, showing anodic peak at 0.975 V.

The suggested method is regarded as the first DPV method for the determination of BOS in plasma. As demonstrated in Fig. 12, the recommended approach is specific because it can reliably identify BOS, with its distinctive peak at the chosen anodic potential (= 0.975 V against Ag/AgCl), at concentrations of (1.0 × 10^− 6^, 5.0 × 10^− 6^, and 2.0 × 10^− 5^ M) devoid of any interference from the plasma matrix. Based on the information in Table 5, the proposed method was successful in producing good recoveries of 100.37 ± 1.704. The obtained results agreed with the claimed contents of BOS.

**Table 5:** Analysis of BOS in spiked human plasma.

Claimed (M)	Found (M)	% Recovery *
1.00 × 10^− 6^	1.02 × 10^− 6^	102.00 ± 1.51
5.00 × 10^− 6^	4.93 × 10^− 6^	98.60 ± 0.84
2.00 × 10^− 5^	2.01 × 10^− 5^	100.50 ± 0.26
Mean ± SD		100.37 ± 1.704

*Average of three determinations.

### Greenness assessment of the proposed method

Greenness of the proposed study was evaluated using two greenness metrics, namely the analytical eco-scale and the AGREE approach. Analytical eco-scale is calculated by deducting penalty points from a base of 100 for each analytical method component. In conformity with its standards, a method that is ideally green scores an eco-scale of 100, an excellent green method scores an eco-scale of more than 75, and an acceptable green method scores an eco-scale of more than 50^34^. Out of all the greenness evaluation techniques, only the AGREE approach uses all 12 green analytical chemistry (GAC) principles and yields an easily interpretable and informative result^35^. The analytical method is considered green for drug analysis if the AGREE analytical score is greater than 0.75. Additionally, a score of 0.50 indicates that the method is acceptably green for drug analysis. Scores less than 0.50 show that the proposed analytical process is unacceptable from the greenness point of view.

According to the analytical eco-scale, the proposed method showed excellent greenness with a score of 78.0. Meanwhile, evaluation using AGREE software yielded a score of 0.65, indicating the acceptable greenness of the proposed method. Orange color was assigned to principles 2, 3, 5, 7, and 11 referring to the sample volume needed in this method (10.0 mL), the performed measurement being in at-line mode, the technique being semi-automated, being not miniaturized, the amount of waste generated is (10.0 mL), and 4.0 mL methanol were used (flammable, and corrosive); respectively. These results are demonstrated in Table 6.

**Table 6:**
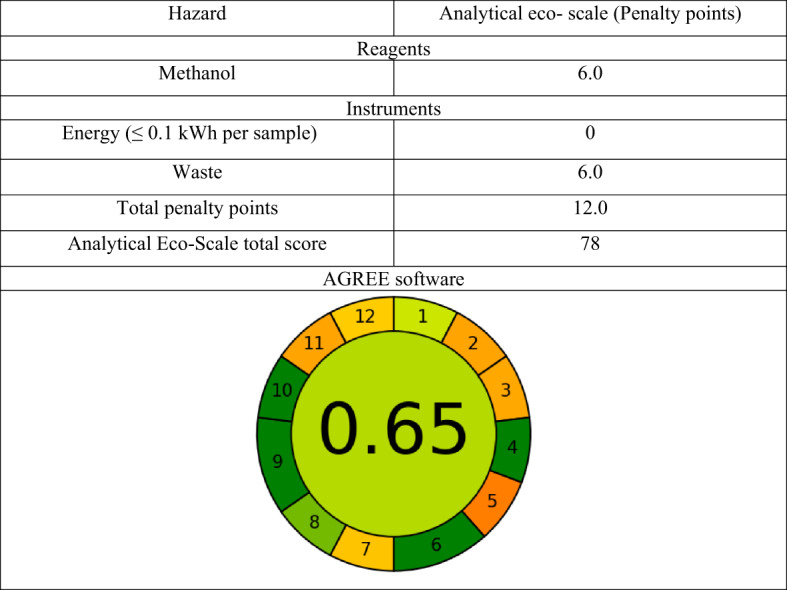
Greenness assessment of the proposed voltammetric method using analytical Eco- scale and AGREE software.

### Statistical analysis of the results

A statistical comparison was made between the results obtained from the proposed approach and the reported one^13^. Upon computation, the t and F values were found to be less than the tabulated values, suggesting the absence of a statistically significant difference. The results are shown in Table 7, and demonstrating that the suggested approach is both exact and accurate.

**Table 7:** Statistical comparison between the proposed method and the reported one for the determination of BOS.

Parameter	Reported method* (13)	Proposed method
Mean	99.83	99.35
SD	0.45	0.88
Variance	0.20	0.77
N	3.0	5.0
Student t-test (2.447)		0.86
F (19.247)		3.85

* Inertsil C8 column (5 µ, 15 cm x 4.6 mm) followed by a guard column CLC ODS (4 cm x 4.6 mm, i.d.) was used for chromatographic separation by isocratic elusion. acetate buffer (pH 5.5) and acetonitrile in the ratio of 20:80 (v/v) were used as mobile phase with a flow of 1.0 mL/min.

## Conclusion

The suggested approach represents the first DPV method using Au-NPsmodified PGE applied to spiked human plasma. Modification with Au-NPs enhanced the sensor’s sensitivity; resulting in a lower limit of detection (LOD) compared to previously reported voltammetric method, enabling its application to human plasma. This approach offers several advantages, including rapid analysis, procedural simplicity, appropriate validation parameters, and the use of cost-effective instrumentation. Demonstrating acceptable accuracy and precision, the method successfully determined BOS in pharmaceutical dosage forms and spiked human plasma without interference from tablet excipients or plasma matrix components.

The method demonstrated satisfactory analytical performance along with strong compliance with green analytical chemistry principles. Greenness assessment using the Analytical Eco-Scale and the AGREE approach confirmed the environmental sustainability of the proposed method, yielding an excellent Eco-Scale score of 78.0 and an acceptable AGREE score of 0.65. These results indicate that the method effectively balances analytical efficiency with environmental responsibility, making it suitable for routine pharmaceutical analysis and quality control applications.

## Study limitations and future prospects

The current study was conducted only on spiked human plasma samples. Future investigations could examine the pharmacokinetics of BOS in plasma samples from human volunteers using the proposed method. Furthermore, extending this method to other biological matrices, such as urine, would be advantageous.

## Supplementary Information

Below is the link to the electronic supplementary material.

Supplementary Material 1

## Data Availability

All data generated or analyzed during this study are included in this published article [and its supplementary information files].
