# Supplementary material for: Sensitive voltammetric detection of bosentan using gold nanoparticles-decorated pencil graphite electrode in pharmaceutical formulations and plasma samples
Source: Sci Rep. 2026 Mar 27;16:10479. doi: 10.1038/s41598-026-42667-w (PMC13031298; doi:10.1038/s41598-026-42667-w)
Supplement: Supplementary file 1 — Supplementary Material 1 [file 41598_2026_42667_MOESM1_ESM.docx]

**Supplementary material for**

**Sensitive Voltammetric Detection of Bosentan Using Gold Nanoparticles‑Decorated Pencil Graphite Electrode in Pharmaceutical Formulations and Plasma Samples**

Yossra A. Trabik*^a^, Reham A. Ismail*^a^, Miriam F. Ayad^a^, Lobna A. Hussein^a^, Amr M. Mahmoud^b^

^a^ Pharmaceutical Analytical Chemistry Department, Faculty of pharmacy, Ain Shams University, Organization of African Unity Street, Abassia 11566, Cairo, Egypt

^b^Analytical Chemistry Department, Faculty of Pharmacy, Cairo University, El-Kasr El-Aini Street, 11562 Cairo, Egypt

*Corresponding authors’ emails: [ytrabik@pharma.asu.edu.eg](mailto:ytrabik@pharma.asu.edu.eg), [rihamabdelaziz.ismail@pharma.asu.edu.eg](mailto:rihamabdelaziz.ismail@pharma.asu.edu.eg)


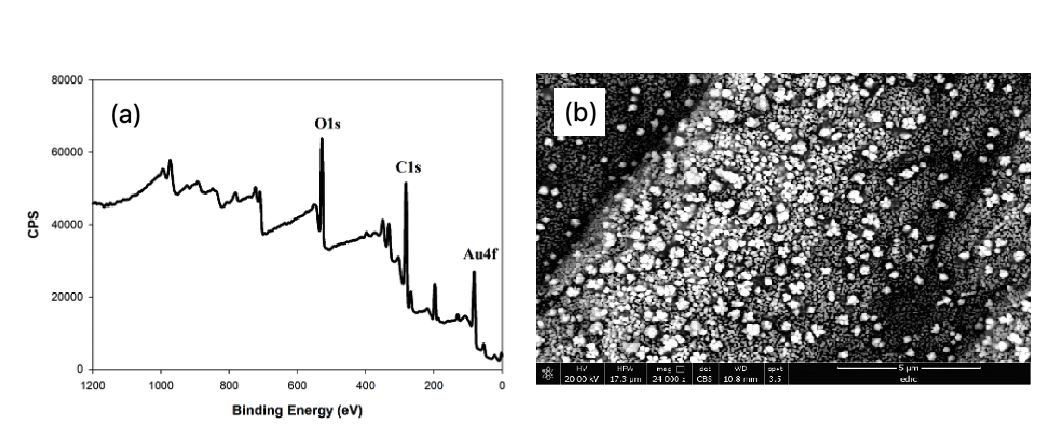


**Figure S1: (a)** X-ray photoelectron spectrum of PGE/Au-NPs electrode, (b) SEM image of PGE/Au-NPs electrode.

**
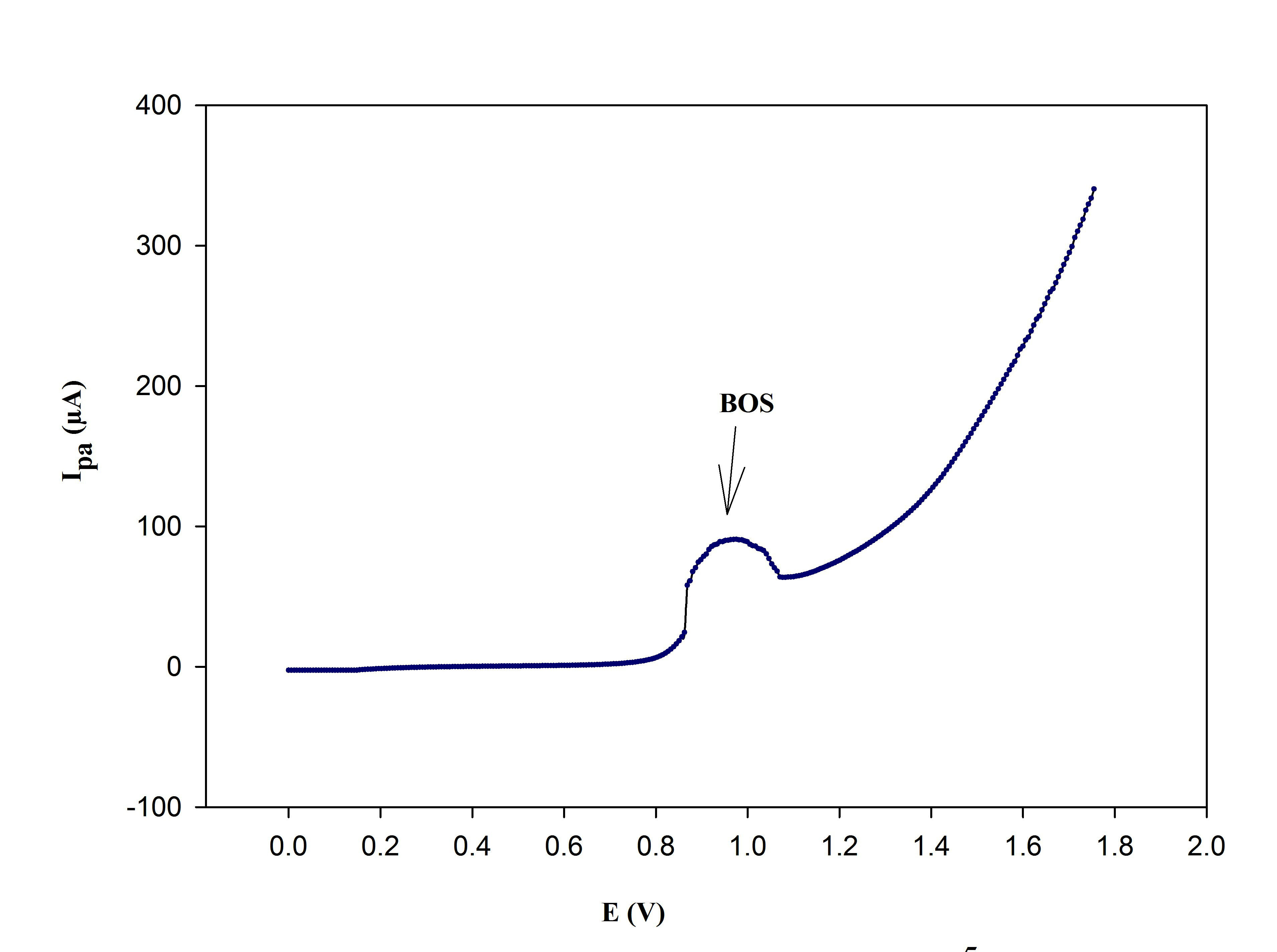
**

**Figure S2:**  Differential pulse voltammogram of 2.5 × 10^-5^ M BOS using acetate buffer as supporting electrolyte at pH=4.5 using PGE/Au-NPs electrode with scan rate of 50 mV/s, showing a broad anodic peak at 0.95 V.


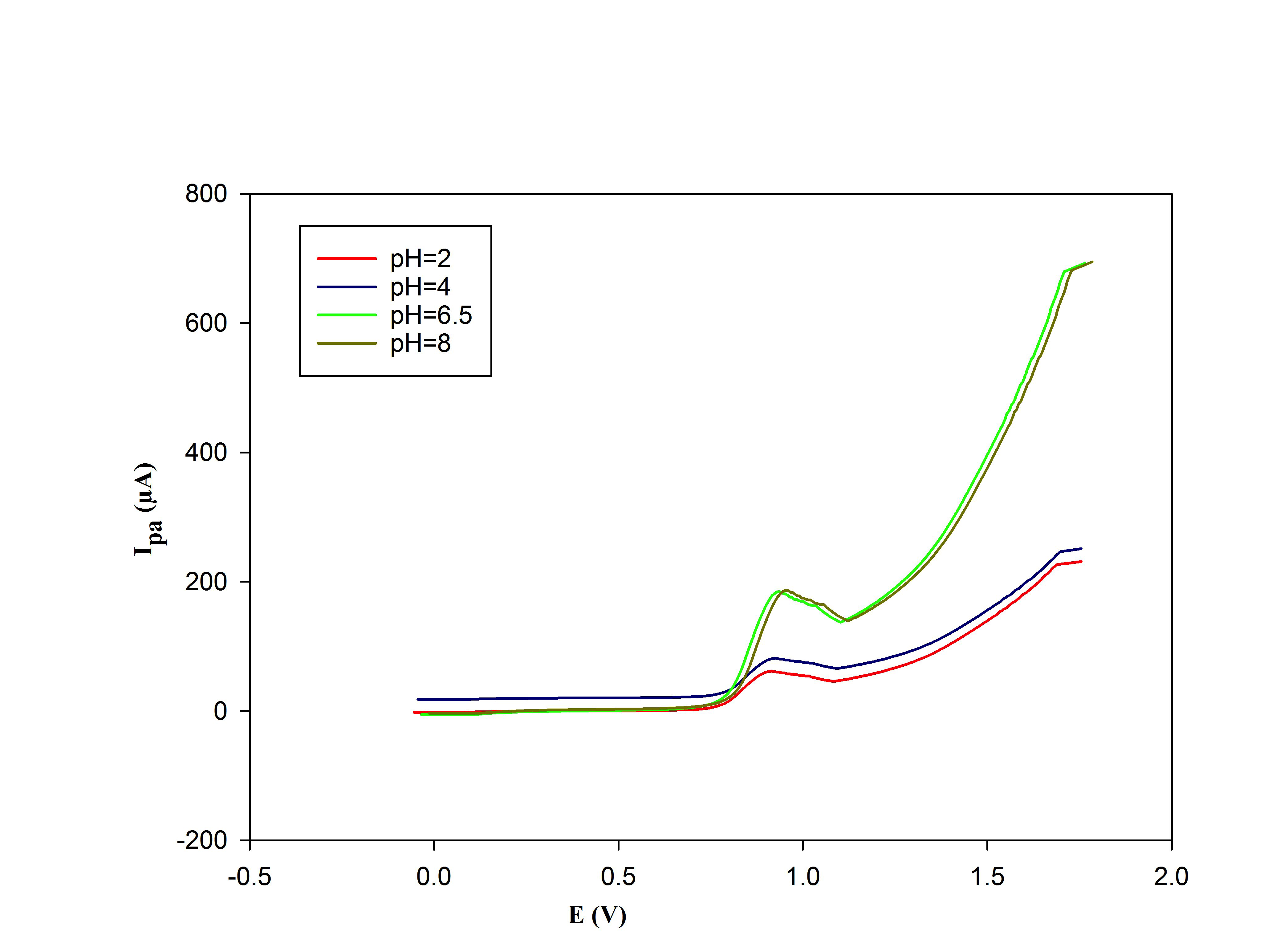


**Figure S3:**  Differential pulse voltammograms of 2.5 × 10^-5^ M BOS using Britton-Robinson buffer as supporting electrolyte at different pHs using PGE/Au-NPs electrode with scan rate of 50 mV/s.

**
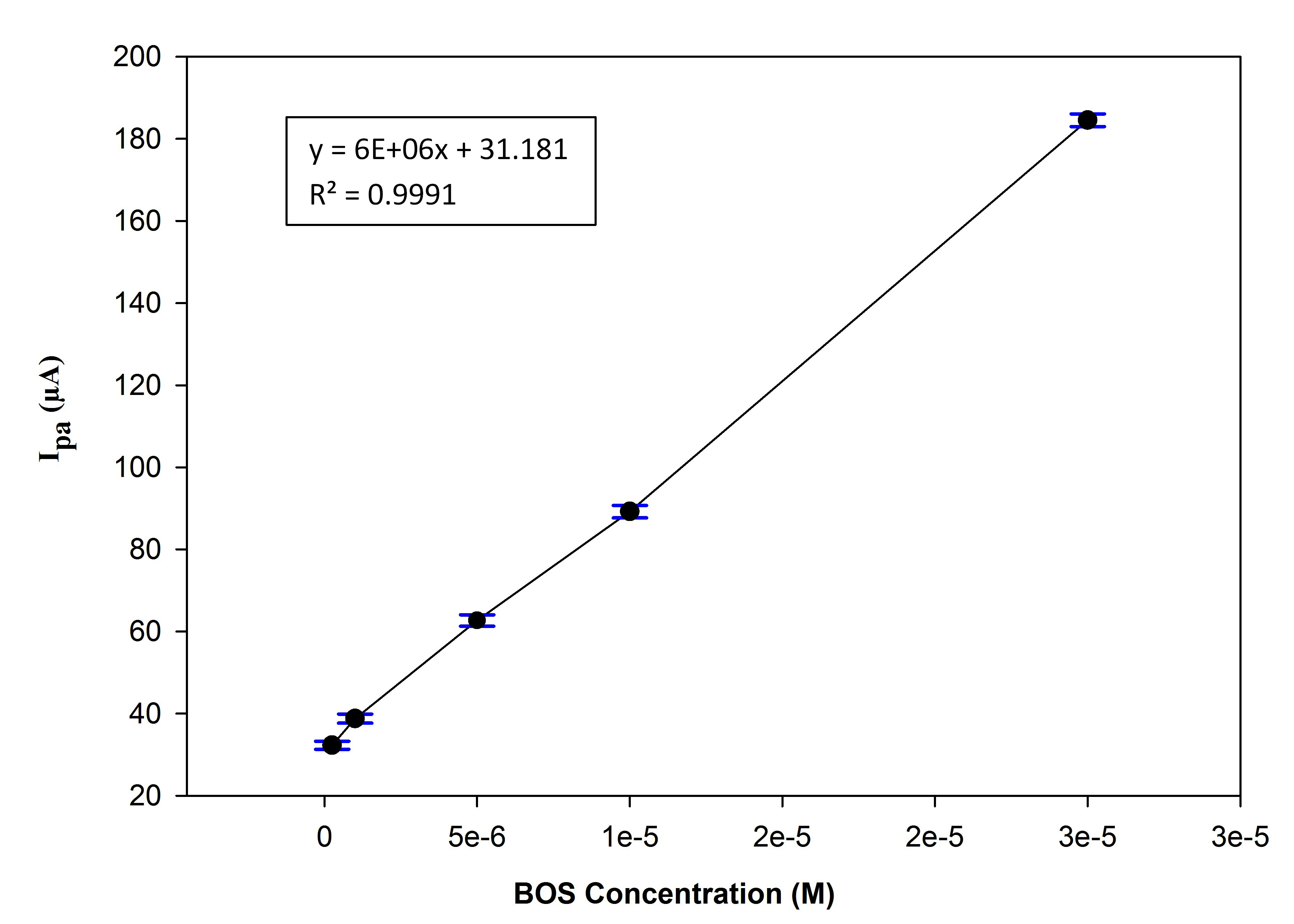
**

**Figure S4:** Calibration curve for determination of BOS in the range of (2.5 x 10^-7^ – 2.5 x 10^-5^ M) using PGE/Au-NPs electrode (with error bars).
